# Risk Factors for Development of Chronic Kidney Disease following Renal Infarction: Retrospective Evaluation of Emergency Room Patients from a Single Center

**DOI:** 10.1371/journal.pone.0098880

**Published:** 2014-06-09

**Authors:** Wen-Ling Lin, Chen-June Seak, Jiunn-Yih Wu, Yi-Ming Weng, Hang-Cheng Chen

**Affiliations:** 1 Department of Emergency Medicine, Chang Gung Memorial Hospital, Taoyuan, Taiwan, and Chang Gung University College of Medicine, Taoyuan, Taiwan; 2 Department of Emergency Medicine, Chang Gung Memorial Hospital, Keelung, Taiwan, and Chang Gung University College of Medicine, Taoyuan, Taiwan; Mario Negri Institute for Pharmacological Research and Azienda Ospedaliera Ospedali Riuniti di Bergamo, Italy

## Abstract

**Background:**

Previous studies have analyzed factors associated with renal infarction so that patients can be provided with earlier diagnosis and treatment. However, the factors associated with development of chronic kidney disease (CKD) following renal infarction are unknown.

**Methods:**

We retrospectively reviewed the records of patients with a diagnosis of renal infarction based on enhanced computed tomography. All patients were admitted to a single emergency department in Taiwan from 1999 to 2008. Univariate and multivariate analysis were used to assess the effect of different factors on development of CKD based on estimates of the glomerular filtration rate (eGFR) at admission and at 3–12 months after discharge.

**Results:**

Univariate analysis indicated significantly increased risk of CKD in patients older than 50 years, with symptoms for 24 h or less before admission, lower eGFR at admission, APACHE II score greater than 7, SOFA score greater than 1, ASA score greater than 2, and SAPS II score greater than 15. Multivariate analysis indicated that only SOFA score greater than 1 was significantly and independently associated with CKD at follow-up (*p*<0.001).

**Conclusions:**

A total of 32.5% of patients admitted for renal infarction over a ten-year period developed CKD at 3–12 months after discharge. A SOFA score greater than 1 was significantly and independently associated with development of CKD in these patients.

## Introduction

Renal infarction is a rare but potentially serious condition that is typically characterized by unilateral flank or abdominal pain, hematuria, and proteinuria. Diagnosis can be difficult because these and other signs and symptoms are non-specific and are often consistent with pyelonephritis, tumor, urolithiasis, and other conditions [1]. Diagnosis is best made by contrast-enhanced computed tomography (CT) and/or renal angiography. CT results typically indicate the presence of a wedge-shaped hypodense area in the peripheral region [2], [3]. Thrombosis, embolism, and arterial lesion are the main causes of renal infarction [4].

Prompt initiation of therapy with an anti-coagulant and/or a thrombolytic agent is often the preferred treatment for renal infarction. Catheter-directed intra-arterial thrombolysis may also be effective [5]. However, such treatments are not effective in all patients, possibly due to delays in diagnosis [2]. Patients can recover from renal infarction, but some patients develop irreversible chronic kidney disease (CKD). Many previous case studies and case series have investigated factors associated with renal infarction in order to allow earlier diagnosis [3]–[8]. However, the factors associated with development of CKD following renal infarction are not yet well-established.

In the present study, we identified factors associated with development of CKD following renal infarction by analysis of patients from a single institution in Taiwan who experienced renal infarction from 1999 to 2008.

## Methods

### Patient selection

All patients were admitted to the Emergency Department (ED) of Chang Gung Memorial Hospital (Taoyuan, Taiwan) from 1999 to 2008 due to a diagnosis of renal infarction based on enhanced computed tomography (CT). All patients received out-patient follow-up and had serum creatinine measured at admission. Variables were excluded if they were missing in more than 50% of patients, if all subjects had the same value, and if they were not numerically coded. Ultimately, 40 patients were enrolled. The Institutional Review Board of Chang Gung Memorial Hospital approved this retrospective study and the patients provided written informed consent.

### Clinical and demographic data

Clinical and demographic data were obtained by retrospective chart review. Serum creatinine was measured by the Jaffe reaction with alkaline picrate. Prognostic scores were calculated upon ED admission (before treatment) based on APACHE II criteria [9], SOFA criteria [10], ASA criteria [11], and SAPS II criteria [12]. These scores were readily available, and although they were not designed to predict the risk of CKD after RI, we investigated their potential use in this new context. CKD was diagnosed based on K/DOQI guidelines [13] and an estimated glomerular filtration rate (eGFR) less than 60 mL/min/1.73 m^2^ at ER admission and 3 months after discharge. The eGFR was calculated by the Cockcroft and Gault formula, which was developed in 1976 [14].

### Data Analysis

Continuous variables are expressed as means ± SDs if the distribution was normal and as medians with interquartile ranges (IQRs) if the distribution was non-normal. Categorical variables are expressed as frequencies and percentages. The difference in eGFR at two time points was assessed by a paired *t*-test. The differences in the presence of CKD in patients with different characteristics were assessed by a Chi-square test or Fisher's exact test, as appropriate. For identification of risk factors associated with CKD, the point estimates and 95% confidence intervals (CIs) of odds ratios (ORs) were calculated by univariate and multivariate logistic regression models. All significant variables in the univariate analysis were used for backward selection in the multivariate logistic regression model; variables that did not improve model fit for a *p*-value less than 0.1 were discarded, but potential confounding variables (age and gender) were forced into the model. All statistical analyses were performed with SAS software version 9.2 (SAS Institute Inc., Cary, NC). A two-tailed *p*-value less than 0.05 was considered statistically significant.

## Results

### Baseline characteristics of study subjects

A total of 40 patients (29 males [72.5%] and 11 females [27.5%]) who were admitted to our ED for renal infarction, all of whom had eGFR measurements at admission and at 3–12 months after discharge, were included in the analysis (Table 1). The mean patient age was 53.18±15.10 years and the age range was 20 to 78 years. One patient (2.5%) had a previous renal infarction. The most common medications were aspirin (35%) and warfarin (15%). The median (IQR) duration of symptoms/signs before admission was 24.0 h (12.0–84.0). The most common symptoms were abdominal pain (85%) and flank pain (42.5%). Eighteen patients (45%) had an embolism in the heart or other region. The median (IQR) of the APACHE II, SOFA, ASA, and SAPS II scores were 7.0 (4.0–10.0), 1.0 (0.0–2.0), 2.0 (1.0–3.0), and 15.0 (13.0–19.0), respectively. Based on a Kruskal-Wallis test, the distributions of ICU prognostic scores (APACHE II, SOFA, ASA, and SAPS II) did not differ significantly among patients with different etiologies (cardiac or other embolism, n = 18; thrombosis or coagulation dysfunction, n = 16; renal artery dissection, n = 1; idiopathic disease, n = 5).

**Table 1:** Baseline characteristics of study subjects admitted to the emergency department for renal infarction (n = 40).

Characteristic	
Age (years), mean ± SD	53.18±15.10
Gender, n (%)	
Female	11 (27.5)
Male	29 (72.5)
Previous renal infarction, n (%)	
No	39 (97.5)
Yes	1 (2.5)
***Current Medications***	
Aspirin, n (%)	
No	26 (65.0)
Yes	14 (35.0)
Warfarin, n (%)	
No	34 (85.0)
Yes	6 (15.0)
Amiodarone, n (%)	
No	39 (97.5)
Yes	1 (2.5)
Clopidogrel, n (%)	
No	40 (100.0)
Yes	0 (0.0)
Steroid, n (%)	
No	39 (97.5)
Yes	1 (2.5)
Chinese herb, n (%)	
No	39 (97.5)
Yes	1 (2.5)
Hormone replacement therapy, n (%)	
No	38 (95.0)
Yes	2 (5.0)
***Clinical symptoms***	
Duration (h) of symptoms/signs before admission, median (IQR)	24.0 (12.0–84.0)
Fever, n (%)	
No	34 (85.0)
Yes	6 (15.0)
Chills, n (%)	
No	39 (97.5)
Yes	1 (2.5)
Abdominal pain, n (%)	
No	6 (15.0)
Yes	34 (85.0)
Abdominal fullness, n (%)	
No	36 (90.0)
Yes	4 (10.0)
Dyspnea, n (%)	
No	36 (90.0)
Yes	4 (10.0)
Flank pain, n (%)	
No	23 (57.5)
Yes	17 (42.5)
Back pain, n (%)	
No	37 (92.5)
Yes	3 (7.5)
Nausea, n (%)	
No	26 (65.0)
Yes	14 (35.0)
Vomiting, n (%)	
No	28 (70.0)
Yes	12 (30.0)
Hematuria, n (%)	
No	39 (97.5)
Yes	1 (2.5)
Oligouria, n (%)	
No	40 (100.0)
Yes	0 (0.0)
Anuria, n (%)	
No	40 (100.0)
Yes	0 (0.0)
Etiology, n (%)	
Cardiac or other embolism	18 (45.0)
Thrombosis or coagulation dysfunction	16 (40.0)
Spontaneous renal artery dissection	1 (2.5)
Idiopathic	5 (12.5)
***Laboratory test***	
eGFR at admission (mL/min per 1.73 m^2^), median (IQR)	66.70±31.03
***ICU prognostic scores***	
APACHE II, median (IQR)	7.0 (4.0–10.0)
SOFA, median (IQR)	1.0 (0.0–2.0)
ASA, median (IQR)	2.0 (1.0–3.0)
SAPS II, median (IQR)	15.0 (13.0–19.0)

### eGFR at admission and 3 to 12 months after discharge

**Figure 1:**
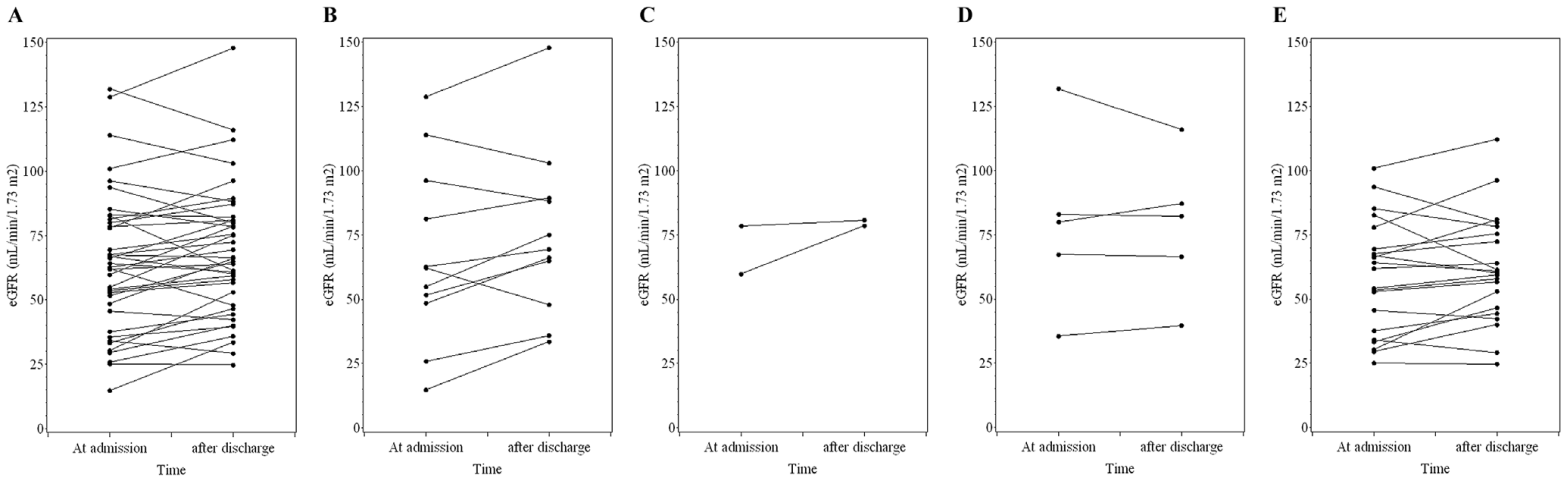
Changes in eGFR for (A) all patients (n = 40), and those evaluated at (B) 3 months after discharge (n = 11), (C) 6 months after discharge (n = 2), (D) 9 months after discharge (n = 6), and (E) 12 months after discharge (n = 21).

The eGFRs of all enrolled patients were measured at admission and 3 to 12 months after discharge (Table 2). Eleven patients were evaluated at month-3, 2 at month-6, 6 at month-9, and 21 at month-12 (mean duration: 8.8±3.9 months after discharge) (Figure 1). The results indicate slightly higher mean eGFR after discharge than at admission (66.7±31.0 *vs.* 70.5±28.5 mL/min/1.73 m^2^, *p* = 0.035), but no significant change in Cr.

**Table 2:** Mean (±SD) eGFR and Cr of patients at admission and at 3 to 12 months after discharge (n = 40).

Parameter	At admission	3 to 12 months after discharge	Difference	*p*-value†
eGFR (mL/min/1.73 m^2^)	66.7±31.0	70.5±28.5	3.8±11.1	0.035
Cr (mg/dL)	1.35±0.64	1.23±0.38	−0.12±0.46	0.115

† Paired t-test.

### Factors associated with development of chronic kidney disease

A total of 13 patients (32.5%) developed CKD at the last follow-up. Table 3 shows a univariate analysis of risk factors associated with development of CKD. These results indicate significantly increased risk of CKD in patients with the following characteristics: older than 50 years (50.0% *vs.* 11.1%, *p* = 0.009), symptoms for 24 h or less before admission (45.8% *vs.* 12.5%, *p* = 0.027), lower eGFR at admission (per 1 mL/min/1.73 m^2^, OR = 0.74, 95% CI: 0.56–0.98, *p* = 0.034), APACHE II score greater than 7 (50.0% *vs.* 18.2%, *p* = 0.033), SOFA score greater than 1 (90.9% *vs.* 10.3%, *p*<0.001), ASA score greater than 2 (71.4% *vs.* 11.5%, *p*<0.001), and SAPS II score greater than 15 (57.9% *vs.* 9.5%, *p* = 0.001).

**Table 3:** Univariate analysis of risk factors associated with chronic kidney disease at follow-up among patients admitted for renal infarction.

Characteristic	CKD/Total (%)	*p*-value	OR (95% CI)	*p*-value
Age (years)				
≦50	2/18 (11.1)	0.009†	1.00 (reference)	
>50	11/22 (50.0)		8.00 (1.48–43.40)	0.016
Gender				
Female	3/11 (27.3)	1.000^‡^	1.00 (reference)	
Male	10/29 (34.5)		1.40 (0.30–6.49)	0.665
Previous renal infarction				
No	13/39 (33.3)	1.000^‡^	1.00 (reference)	
Yes	0/1 (0.0)		NA	0.981
***Current Medications***				
Aspirin				
No	6/26 (23.1)	0.155^‡^	1.00 (reference)	
Yes	7/14 (50.0)		3.33 (0.83–13.37)	0.089
Warfarin				
No	11/34 (32.4)	1.000^‡^	1.00 (reference)	
Yes	2/6 (33.3)		1.05 (0.17–6.61)	0.962
Amiodarone				
No	13/39 (33.3)	1.000^‡^	1.00 (reference)	
Yes	0/1 (0.0)		NA	0.981
Steroid				
No	12/39 (30.8)	0.325^‡^	1.00 (reference)	
Yes	1/1 (100.0)		NA	0.982
Chinese herb				
No	13/39 (33.3)	1.000^‡^	1.00 (reference)	
Yes	0/1 (0.0)		NA	0.981
Hormone therapy				
No	12/38 (31.6)	1.000^‡^	1.00 (reference)	
Yes	1/2 (50.0)		2.17 (0.13–37.64)	0.596
***Clinical symptoms***				
Duration (h) of symptoms/signs before admission				
≦24^a^	11/24 (45.8)	0.027†	1.00 (reference)	
>24	2/16 (12.5)		0.17 (0.03–0.91)	0.039
Fever				
No	12/34 (35.3)	0.643^‡^	1.00 (reference)	
Yes	1/6 (16.7)		0.37 (0.04–3.51)	0.384
Chills				
No	13/39 (33.3)	1.000^‡^	1.00 (reference)	
Yes	0/1 (0.0)		NA	0.981
Abdominal pain				
No	0/6 (0.0)	0.152^‡^	1.00 (reference)	
Yes	13/34 (38.2)		NA	0.952
Abdominal fullness				
No	12/36 (33.3)	1.000^‡^	1.00 (reference)	
Yes	1/4 (25.0)		0.67 (0.06–7.11)	0.737
Dyspnea				
No	11/36 (30.6)	0.584^‡^	1.00 (reference)	
Yes	2/4 (50.0)		2.27 (0.28–18.27)	0.440
Flank pain				
No	5/23 (21.7)	0.091†	1.00 (reference)	
Yes	8/17 (47.1)		3.20 (0.81–12.65)	0.097
Back pain				
No	12/37 (32.4)	1.000 ^‡^	1.00 (reference)	
Yes	1/3 (33.3)		1.04 (0.09–12.66)	0.974
Nausea				
No	9/26 (34.6)	1.000^‡^	1.00 (reference)	
Yes	4/14 (28.6)		0.76 (0.18–3.11)	0.698
Vomiting				
No	9/28 (32.1)	1.000^‡^	1.00 (reference)	
Yes	4/12 (33.3)		1.06 (0.25–4.45)	0.941
Hematuria				
No	13/39 (33.3)	1.000^‡^	1.00 (reference)	
Yes	0/1 (0.0)		NA	0.981
Etiology				
Cardiac or other embolism	8/18 (44.4)	0.379^‡^	1.20 (0.16–9.01)	0.966
Thrombosis or coagulation dysfunction	3/16 (18.8)		0.35 (0.04–3.08)	0.980
Spontaneous renal artery dissection	0/1 (0.0)		NA	0.971
Idiopathic	2/5 (40.0)		1.00 (reference)	
***Laboratory test***				
eGFR at admission, per 1 ml/min/1.73 m^2^			0.74 (0.56–0.98)	0.034
***ICU prognostic scores***				
APACHE II				
≦7^a^	4/22 (18.2)	0.033†	1.00 (reference)	
>7	9/18 (50.0)		4.50 (1.08–18.69)	0.038
SOFA				
≦1^a^	3/29 (10.3)	<0.001^‡^	1.00 (reference)	
>1	10/11 (90.9)		86.67 (8.04–934.35)	<0.001
ASA				
≦2^a^	3/26 (11.5)	<0.001^‡^	1.00 (reference)	
>2	10/14 (71.4)		19.17 (3.61–101.91)	0.001
SAPS II				
≦15^a^	2/21 (9.5)	0.001†	1.00 (reference)	
>15	11/19 (57.9)		13.06 (2.34–72.82)	0.003
***Treatments***				
Drug therapy				
None	4/16 (25.0)	0.647^‡^	1.00 (reference)	
coumadin	0/2 (0.0)		NA	0.958
unfractioned heparin	4/9 (44.4)		2.40 (0.42–13.60)	0.936
LMWH	4/9 (44.4)		2.40 (0.42–13.60)	0.936
coumadin+UFH	0/1 (0.0)		NA	0.969
coumadin+LMWH	1/1 (100.0)		NA	0.932
coumadin+UFH+LMWH	0/1 (0.0)		NA	0.969
UFH+TPA(IV)	0/1 (0.0)		NA	0.969
Surgery				
No	13/36 (36.1)	0.284^‡^	1.00 (reference)	
Yes	0/4 (0.0)		NA	0.961
Blood pressure control				
No	10/32 (31.3)	1.000^‡^	1.00 (reference)	
Yes	3/8 (37.5)		1.32 (0.26–6.64)	0.736
Emergent dialysis				
No	12/39 (30.8)	0.325^‡^	1.00 (reference)	
Yes	1/1 (100.0)		NA	0.979
Poor response to opioid				
No	10/29 (34.5)	1.000^‡^	1.00 (reference)	
Yes	3/11 (27.3)		0.71 (0.15–3.30)	0.665
Pain subsided				
No	6/22 (27.3)	0.435†	1.00 (reference)	
Yes	7/18 (38.9)		1.70 (0.45–6.44)	0.437
***Type of renal infarction***				
Left renal infarction				
No	9/21 (42.9)	0.142†	1.00 (reference)	
Yes	4/19 (21.1)		0.36 (0.09–1.44)	0.148
Right renal infarction				
No	8/27 (29.6)	0.722^‡^	1.00 (reference)	
Yes	5/13 (38.5)		1.48 (0.37–5.96)	0.577
Bilateral renal infarction				
No	9/32 (28.1)	0.400^‡^	1.00 (reference)	
Yes	4/8 (50.0)		2.56 (0.52–12.48)	0.246
Splenic infarction				
No	11/34 (32.4)	1.000^‡^	1.00 (reference)	
Yes	2/6 (33.3)		1.05 (0.17–6.61)	0.962
***Complications***				
ESRD				
No	5/21 (23.8)	0.217†	1.00 (reference)	
Yes	8/19 (42.1)		2.33 (0.60–9.03)	0.222
Hospital Stay (day)				
≦10^a^	13/37 (35.1)	0.538^‡^	1.00 (reference)	
>10	0/3 (0.0)		NA	0.967
ICU Stay (day)				
≦0^a^	13/38 (34.2)	1.000^‡^	1.00 (reference)	
>0	0/2 (0.0)		NA	0.973
Ventilator use				
No	2/18 (11.1)	0.009†	1.00 (reference)	
Yes	11/22 (50.0)		8.00 (1.48–43.40)	0.016

Abbreviations: OR, odds ratio; CI, confidence interval; NA, non-available; CKD, chronic kidney disease; ESRD, end stage renal disease; LMWH, low molecular weight heparin; TPA, tissue plasminogen activator; UFH, unfractionated heparin.

† Chi-square test; ^‡^Fisher's exact test.

a Median served as the cutoff value.

The final multivariate model was chosen with backward selection for variables that were significant in the univariate analysis. Variables that did not improve model fit (*p*<0.1) were discarded, except for age and gender, which were forced in the model for adjustment (Table 4). The results of this analysis indicated that only SOFA score greater than 1 was significantly and independently associated with CKD at follow-up (adjusted OR = 75.60, 95% CI: 5.62–1016.99, *p* = 0.001). In addition, previous hypertension and diabetes mellitus were not significantly associated with CKD (data not shown).

**Table 4:** Multivariate analysis of risk factors associated with chronic kidney disease at follow-up among patients admitted for renal infarction.

Characteristic	Adjusted OR (95% CI)	*p*-value
Age (years)		
≦50	1.00 (reference)	
>50	7.12 (0.54–93.40)	0.135
Gender		
Female	1.00 (reference)	
Male	1.23 (0.11–14.23)	0.868
SOFA		
≦1^a^	1.00 (reference)	
>1	75.60 (5.62–1016.99)	0.001

OR: odds ratio; CI: confidence interval.

a Median served as the cutoff value.

## Discussion

We examined the records of 40 patients who were admitted to the ED of Chang Gung Memorial Hospital from 1999 to 2008 with a diagnosis of renal infarction based on enhanced contrast CT. Measurements indicate slightly higher eGFR after discharge than at admission, suggesting a slight improvement in renal function after discharge. A total of 13 patients (32.5%) with renal infarction were diagnosed with CKD after discharge. Univariate analysis indicated that numerous factors were significantly associated with CKD (age >50 years, symptoms for 24 h or less before admission, low eGFR at admission, APACHE II score greater than 7, SOFA score greater than 1, ASA score greater than 2, and SAPS II score greater than 15. However, multivariate analysis indicated that only SOFA score greater than 1 was significantly and independently associated with a diagnosis of CKD. Although the SOFA score was not designed to predict the risk of CKD after RI, our results suggest a potential new use for this parameter.

It may seem somewhat surprising that our multivariate analysis indicated that SOFA score was independently associated with development of CKD, but that the APACHE II, SAPS II, and ASA scores were not. The SOFA score is an indicator of organ dysfunction and morbidity that was originally developed to assess organ dysfunction in patients with sepsis, but has been validated for assessment of organ dysfunction in general. Although SOFA score is associated with mortality, the APACHE II, SAPS II, and ASA scores place greater emphasis on prediction of mortality. The authors designed the SOFA system with an emphasis on bedside applicability and simplicity based on commonly available variables. These prognostic metrics are widely used to predict outcomes of patients admitted to intensive care units (ICUs) [15]. Although a patient's individual prognostic score is not dispositive, it is often considered in guiding individual treatment decisions and in the distribution of limited medical resources [16]. Moreover, APACHE II, SAPS II, and ASA scores are often more suitable for characterization of patients with diseases that are imminently life-threatening, or the level of anaesthetic risk. Although renal infarction and CKD are serious conditions, appropriate clinical management allows patients to survive.

Our analysis indicated that the post-discharge eGFR was significantly higher than the eGFR at admission. However, it seems doubtful whether this small increase (3.8±11.1 mL/min/1.73 m^2^) was clinically significant. Nonetheless, these results indicate an improvement in kidney function, so this result is not surprising [17]. We find it encouraging that the SOFA score is the best predictor of CKD, because this score only requires measurement of 6 parameters (respiratory, cardiovascular, central nervous system, renal, coagulation, and liver). The APACHE II requires measurement of 34 variables and SAPS II requires measurement of 12 variables. We suggest that future studies of the development of CKD in patients admitted with RI consider use of the SOFA score to guide patient follow-up.

Our study had several limitations. First, our sample size was relatively small and all patients were from a single institution. Second, all patients were admitted to the ED and exclusion of out-patients with renal infarction may have provided a biased view of factors associated with development of CKD.

## Conclusions

Our retrospective study of patients admitted to a single ED with renal infarction indicated that 32.5% of patients developed CKD within 3 months of discharge. Elevation of serum creatinine at admission and a SOFA score greater than 1? was the factor significantly and independently associated with development of CKD at follow-up.
